# 

**Published:** 1888-08-18

**Authors:** 


CONTENTS.

**A Medical Olive Branch** - 317

**The Seaside Season** - 318

**The Doctor's Pulpit.**—Heat-stroke - 319

**Fever and Cholera Precautions.** By Dr. GEORGE BUCHANAN, F.R.S. - 320

**Turning Over New Leaves** - 321

**A New Surgical Operation** - 322

**Round About the Asylums.**—XII. - 323

**Notes and News** - 324

**Everybody's Page** - 326

**The Student's Column.**—University of Edinburgh - 328

**Annotations.**—The British Medical Association.—The Women's Jubilee Offering - 327

**The Children's Convalescent Home at Great Yarmouth** - 328

**An Ishmaelite.** By LESLIE KEITH, Author of "The Chilcotes," "East and West," etc. - 329

**An Indian Woman's Tribute to Lady Dufferin** - 330

**Doctors and Patients.** By Sir WILLIAM MOORE, K.C.I.E. - 331

**Amusements with Profit for all Ages.**—For Men and Women.—Children's Corner.—Puzzles, etc. - 332

**EXTRA SUPPLEMENT.—THE NURSING MIRROR.**

En Passant.—London School of Medicine for Women.—Elizabeth Fry.—Florence Nightingale.—An Irish Island—Irish Economy.—A Necessary Virtue.—The Police Courts, etc. lxxvii
Original Articles.—A Nurse's Progress.—From Application to Certification.—Paper II. Probation - lxxviii
Everybody's Opinion.—Private Nursing.—Zenana Medical College lxxix
Miss John's Nurses' Home, Glasgow - lxxx
The Nurses' Bookshelf.—Home Nursing.—Diet for the Sick lxxx
Appointments.—Notes and Queries - lxxx
Vacancies - lxxx

